# Supplementary material for: Validation of picogram- and femtogram-input DNA libraries for microscale metagenomics
Source: PeerJ. 2016 Sep 22;4:e2486. doi: 10.7717/peerj.2486 (PMC5036114; doi:10.7717/peerj.2486)
Supplement: Supplemental Information 1 [file peerj-04-2486-s001.pdf]

SUPPL TABLES

Table S1 | Composition of the microbial mock community. The community comprises 54 bacterial and archaeal members with a GC content ranging from 28 to 70%., and relative abundances (Ab.) between 0.04 and 25%. The source (S). is given as Australian Centre for Ecogenomics (ACE), Mircea Podar's lab (M.P), and the Commonwealth Scientific and Industrial Research Organisation CSIRO).

ID	Organism	NCBI Reference	S.	Phylum	%GC	Ab.	G.s.
1	*Escherichia coli* str. K-12 substr. DH10B	NC_010473	ACE	Proteobacteria	51	25.00	4,686,137
2	*Burkholderia* sp. A2	SAMN05213932	ACE	Proteobacteria	67	20.00	7,510,003
3	*Rhizobium* sp.	SAMN05213933	ACE	Proteobacteria	59	6.21	7,162,087
4	*Acinetobacter* sp. YK3	SAMN05213934	ACE	Proteobacteria	41	3.30	3,765,384
5	*Methanococcus maripaludis* C5	NC_009135.1	M.P.	Euryarchaeota	33	3.30	1,780,761
6	*Methanosphaera stadtmanae* MCB3	NC_007681.1	CSIRO	Euryarchaeota	28	2.93	1,556,477
7	*Nanoarchaeum equitans* Kin4-M	NC_005213.1	M.P.	Nanoarchaeota	32	2.93	490,885
8	*Hydrogenobaculum* sp. Y04AAS1	NC_011126.1	M.P.	Aquificae	35	2.29	1,559,514
9	*Bacillus* str. A9	SAMN05225334	ACE	Firmicutes	38	2.29	5,111,925
10	*Enterococcus faecalis* V583	NC_004668.1	M.P.	Firmicutes	38	1.82	3,218,031
11	*Thermus thermophilus* HB8	NC_006461.1	M.P.	Deinococcus-Thermus	70	1.82	1,849,742
12	*Sulfitobacter sp.* NAS-14.1	NZ_AALZ00000000.1	M.P.	Proteobacteria	60	1.36	4,010,516
13	*Chlorobium limicola* DSM 245	NC_010803.1	M.P.	Chlorobi	51	1.36	2,763,181
14	*Methanosarcina* sp. A14	this study	CSIRO	Euryarchaeota	39	1.28	4,275,962
15	*Chlorobium tepidum* TLS	NC_002932.3	M.P.	Chlorobi	57	1.28	2,154,946
16	Chlorobium phaeobacteroides DSM 266	NC_008639.1	M.P.	Chlorobi	48	1.19	3,133,902
17	*Nitrosomonas europaea* ATCC 19718	NC_004757.1	M.P.	Proteobacteria	51	1.19	2,812,094
18	*Archaeoglobus fulgidus* DSM 4304	NC_000917.1	M.P.	Euryarchaeota	49	1.09	2,178,400
19	*Herpetosiphon aurantiacus* ATCC 23779	NC_009972.1	M.P.	Chloroflexi	51	1.09	6,346,587
20	*Thermoanaerobacter pseudethanolicus* ATCC 33223	NC_010321.1	M.P.	Firmicutes	35	0.98	2,362,816
21	*Pyrobaculum calidifontis* JCM 11548	NC_009073.1	M.P.	Crenarchaeota	57	0.98	2,009,313
22	*Pyrobaculum aerophilum* str. IM2	NC_003364.1	M.P.	Crenarchaeota	51	0.89	2,222,430
23	*Thermotoga petrophila* RKU-1	NC_009486.1	M.P.	Thermotogae	46	0.89	1,823,511
24	*Thermotoga neapolitana* DSM 4359	NC_011978.1	M.P.	Thermotogae	47	0.88	1,884,562
25	*Deinococcus radiodurans R1*	NC_001264.1, NC_001263.1	M.P.	Deinococcus-Thermus	67	0.88	3,060,986
26	*Persephonella marina* EX-H1	NC_012440.1	M.P.	Aquificae	37	0.82	1,930,284
27	*Methanobrevibacter* sp. A27	SAMN05224565	CSIRO	Euryarchaeota	51	0.82	1,809,869
28	*Salinispora tropica* CNB-440	NC_009380.1	M.P.	Actinobacteria	69	0.79	5,183,331
29	*Bacteroides vulgatus* ATCC 8482	NC_009614.1	M.P.	Bacteroidetes	42	0.79	5,163,189
30	*Treponema denticola*	NC_002967.9	M.P.	Spirochaetes	38	0.70	2,843,201
31	*Geobacter sulfurreducens* PCA	NC_002939.5	M.P.	Proteobacteria	61	0.70	3,814,128
32	*Caldicellulosiruptor bescii* DSM 6725	NC_012034.1	M.P.	Firmicutes	35	0.63	2,919,718
33	*Chlorobium phaeovibrioides* DSM 265	NC_009337.1	M.P.	Chlorobi	32	0.63	1,966,858
34	*Clostridium thermocellum* ATCC 27405	NC_009012.1	M.P.	Firmicutes	39	0.52	3,843,301
35	*Sulfurihydrogenibium* sp. YO3AOP1	NC_010730.1	M.P.	Aquificae	32	0.52	1,838,442
36	*Shewanella baltica* OS185	NC_009665.1	M.P.	Proteobacteria	46	0.45	5,229,686
37	*Salinispora arenicola* CNS-205	NC_009953.1	M.P.	Actinobacteria	70	0.45	5,786,361
38	*Porphyromonas gingivalis* ATCC 33277	NC_010729.1	M.P.	Bacteroidetes	48	0.44	2,354,886
39	*Methanobacterium* sp. A39	SAMN05224566	CSIRO	Euryarchaeota	33	0.44	3,329,516
40	*Sulfolobus tokodaii* str. 7	NC_003106.2	M.P.	Crenarchaeota	33	0.41	2,694,756
41	*Pyrococcus horikoshii* OT3	NC_000961.1	M.P.	Euryarchaeota	42	0.41	1,738,505
42	*Bordetella bronchiseptica* RB50	NC_002927.3	M.P.	Proteobacteria	68	0.41	5,339,179
43	*Gemmatimonas aurantiaca* T-27	NC_012489.1	M.P.	Gemmatimonadetes	64	0.41	4,636,964
44	*Methanocaldococcus jannaschii* DSM 2661	NC_000909.1	M.P.	Euryarchaeota	31	0.32	1,664,970
45	*Chloroflexus aurantiacus* J-10-fl	NC_010175.1	M.P.	Chloroflexi	57	0.32	5,258,541
46	*Bacteroides thetaiotaomicron* VPI-5482	AE015928.1	M.P.	Bacteroidetes	43	0.30	6,260,361
47	*Rhodopirellula baltica* SH 1	NC_005027.1	M.P.	Planctomycetes	55	0.30	7,145,576
48	*Methanopyrus kandleri* AV19	NC_003551.1	M.P.	Euryarchaeota	61	0.25	1,694,969
49	*Nostoc* sp. PCC 7120	NC_003272.1	M.P.	Cyanobacteria	41	0.25	6,413,771
50	*Bacillus cereus* str. A50	SAMN05231870	CSIRO	Firmicutes	38	0.22	5,372,944
52	*Burkholderia xenovorans* LB400	NC_007951.1,NC_007953.1,NC_007952.1	M.P.	Proteobacteria	63	0.10	9,731,138
53	*Acidobacterium capsulatum* ATCC 51196	NC_012483.1	M.P.	Acidobacteria	61	0.10	4,127,356
54	*Methanobrevibacter smithii* PS	NC_009515.1	CSIRO	Euryarchaeota	31	0.04	1,712,240
55	*Leptothrix cholodnii* SP-6	NC_010524.1	M.P.	Proteobacteria	69	0.04	4,909,403

Table S2. | Analysis of Variance and post-hoc tests. Correlations of mock community member abundances. ANOVA (Analysis of Variance) was performed with MYSTAT and the post-hoc test with SYSTAT. All stats include the 1ng SOP (variable 1) and all low input libraries (100pg, 10pg, 1pg, 100fg; variable 2 to 5) of the mock community as shown in Fig. 2. **(a)** insert size distribution, **(b)** GC-content. Per definition, a significant p-value (<0.05) indicates that the samples are correlated.

(a)

Analysis of Variance

Source	Type III SS	df	Mean Squares	F-ratio	p-value
VAR_1	22,832.356	4	5,708.089	5.445	0.010
Error	12,579.147	12	1,048.262		

Tukey's Honestly-Significant-Difference Test

VAR_2(i)	VAR_2(j)	Difference	p-value	95.0% Confidence Interval	
				Lower	Upper
1	2	50.133	0.369	-34.130	134.396
1	3	53.633	0.310	-30.630	137.896
1	4	88.633	0.038	4.370	172.896
1	5	103.567	0.007	28.200	178.934
2	3	3.500	1.000	-80.763	87.763
2	4	38.500	0.606	-45.763	122.763
2	5	53.433	0.223	-21.934	128.800
3	4	35.000	0.683	-49.263	119.263
3	5	49.933	0.276	-25.434	125.300
4	5	14.933	0.967	-60.434	90.300

(b)

Analysis of Variance

Source	Type III SS	df	Mean Squares	F-ratio	p-value
VAR_1	54.462	4	13.615	3.466	0.050
Error	39.287	10	3.929		

Table S3. | Correlations of mock community member abundances. The Pearson correlation coefficient and the Bonferroni probability (p-value) is shown comparing the 1ng SOP and all low input libraries of the mock community. **(a)** Data set (LIB) including all 54 mock community members, **(b)** data set (LOWAB) excluding the 10 most abundant community members. The correlations and the probability analysis are based on the average relative abundances of all libraries except for one 100fg (re3) which showed a high level of contamination and was therefore excluded, leaving 4 replicates (N4) for the 100 fg libraries.

(a)

Pearson Correlation Matrix

	LIB_1NG	LIB_100PG	LIB_10PG	LIB_1PG	LIB_100FG_N4
LIB_1NG	1.000				
LIB_100PG	0.996	1.000			
LIB_10PG	0.975	0.988	1.000		
LIB_1PG	0.994	0.998	0.992	1.000	
LIB_100FG_N4	0.971	0.984	0.999	0.991	1.000

Matrix of Bonferroni Probabilities

	LIB_1NG	LIB_100PG	LIB_10PG	LIB_1PG	LIB_100FG_N4
LIB_1NG	0.000				
LIB_100PG	0.000	0.000			
LIB_10PG	0.000	0.000	0.000		
LIB_1PG	0.000	0.000	0.000	0.000	
LIB_100FG_N4	0.000	0.000	0.000	0.000	0.000

(b)

Pearson Correlation Matrix

	LOWAB_1NG	LOWAB_100PG	LOWAB_10PG	LOWAB_1PG	LOWAB_100FG_N4
LOWAB_1NG	1.000				
LOWAB_100PG	0.969	1.000			
LOWAB_10PG	0.946	0.943	1.000		
LOWAB_1PG	0.909	0.873	0.934	1.000	
LOWAB_100FG_N4	0.846	0.803	0.923	0.959	1.000

Matrix of Bonferroni Probabilities

	LOWAB_1NG	LOWAB_100PG	LOWAB_10PG	LOWAB_1PG	LOWAB_100FG_N4
LOWAB_1NG	0.000				
LOWAB_100PG	0.000	0.000			
LOWAB_10PG	0.000	0.000	0.000		
LOWAB_1PG	0.000	0.000	0.000	0.000	
LOWAB_100FG_N4	0.000	0.000	0.000	0.000	0.000

Table S4 | Comparison of low, medium and high %GC organisms between libraries. The K-S test for normality showed that the data distribution is significant different from a normal distribution, thus the non-parametric Kruskal-Wallis One-way Analysis of Variance was applied. Each data set included the groups SOP, 100pg, 10pg, 1pg, and 100fg. The observed p-values are not significant (>0.05). Per definition, p-values <0.05 would indicate that the samples are significantly different.

data set	n per group	p-value
low %GC organisms	19	0.855
medium %GC organisms	23	0.940
high %GC organisms	12	0.449

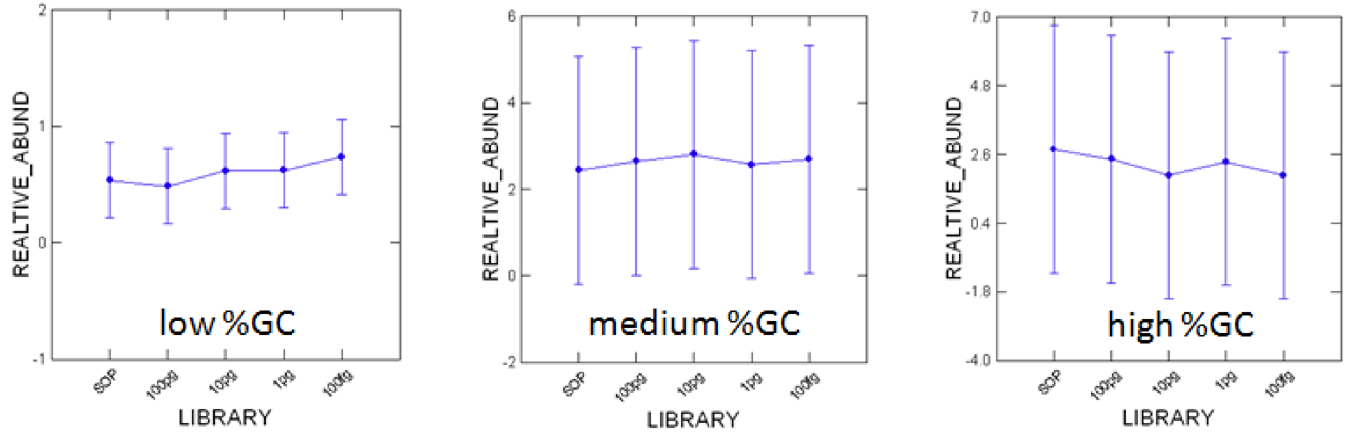

Table S5 | Marine surface water sample population bins. Read were subsample to 50 million reads, and only contigs ≥ 1kb were used for population genome binning. Completeness and contamination were estimated based on marker genes, see Material and Methods. Only bins with a completeness over 50% and a contamination under 10% were included.

	assembly			Completeness (%)			Contamination (%)		
	size	max contig	bins	mean	max	min	mean	max	min
**10L_SOP**	120,202,041	98110	5	66.7	75.5	50.7	2.2	3.0	0.9
**10L_50pg**	120,309,696	104051	4	61.1	69.4	52.3	2.6	3.4	1.6
**10L_5pg**	80,681,654	91557	4	56.8	69.1	52.2	4.1	9.1	0.0
**1ml**	26,440,895	113371	0	0.0	0.0	0.0	0.0	0.0	0.0
**100ul**	49,802,814	45478	0	0.0	0.0	0.0	0.0	0.0	0.0

SUPPL FIGURES

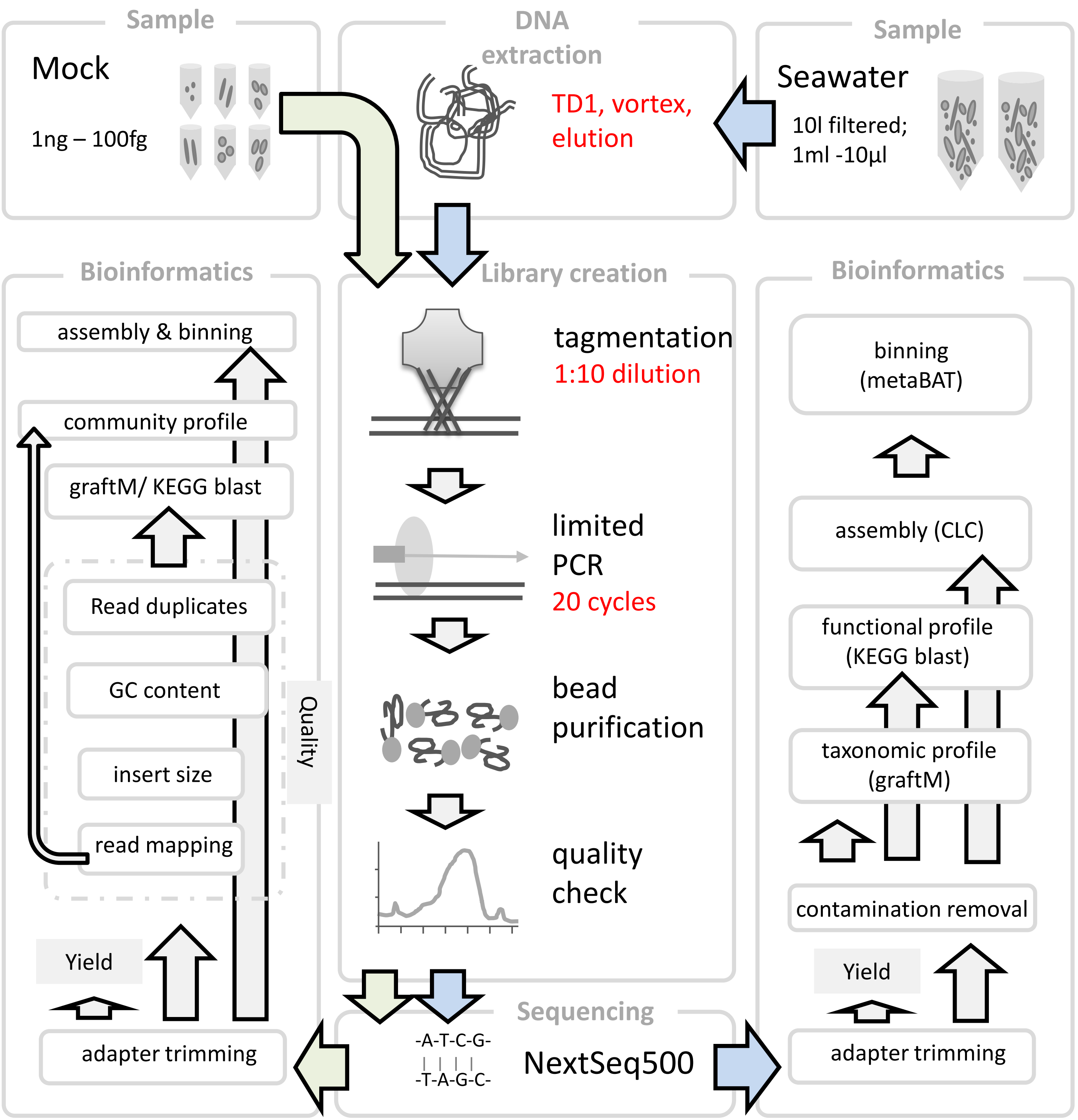

Figure S1 | Sample preparation and analysis workflow with key changes to standard methodology highlighted in red. Mock community samples were obtained as genomic DNA. The DNA extraction of seawater samples was performed according to the manufacturer's protocol (UltraClean® Tissue & Cells) with three main modifications regarding the TD1 lysis solution volume, the homogenizing step, and the volume and incubation time of the elution buffer. Library creation was performed following the manufacturer's protocol (Nextera XT DNA Sample Prep Kit), except for a dilution of the tagmentation reaction (1:10), and an increase in the number of cycles in the limited PCR step (20 cycles). The mock community and seawater sample sequences were adapter trimmed and analyzed with the illustrated bioinformatic pipelines. Green arrows show the main steps for mock community samples, blue arrows show the main steps for seawater samples.

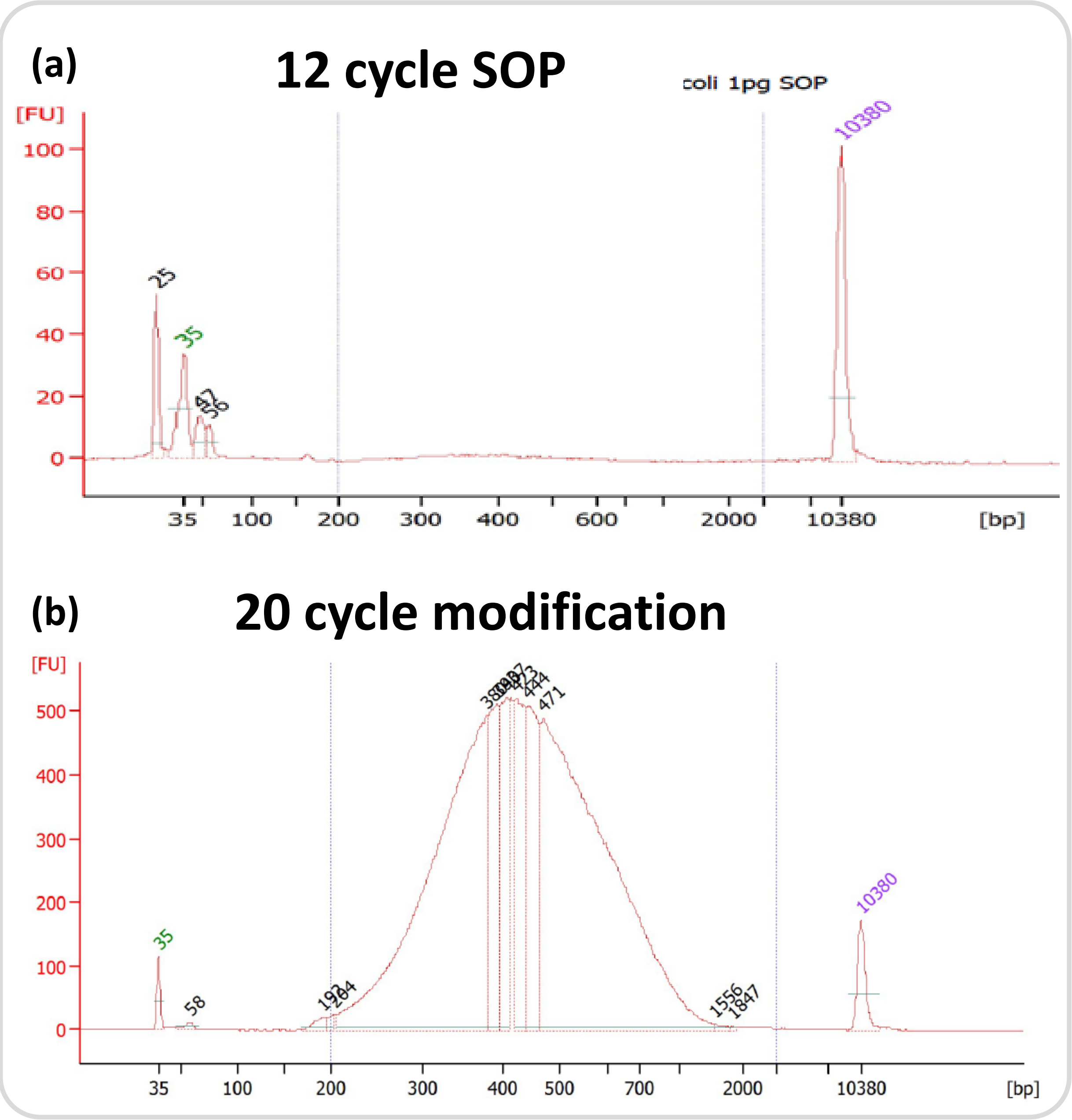

Figure S2 | Comparison between the SOP with 12 cycles of limited cycle PCR and the modified protocol with 20 cycles, tested on 1pg *E. coli* gDNA. The SOP (a) produced no amplification product when evaluated with the Bioanalyzer, whereas the modified 20 cycle protocol (b) yielded the desired product at an amount sufficient for further downstream analysis.

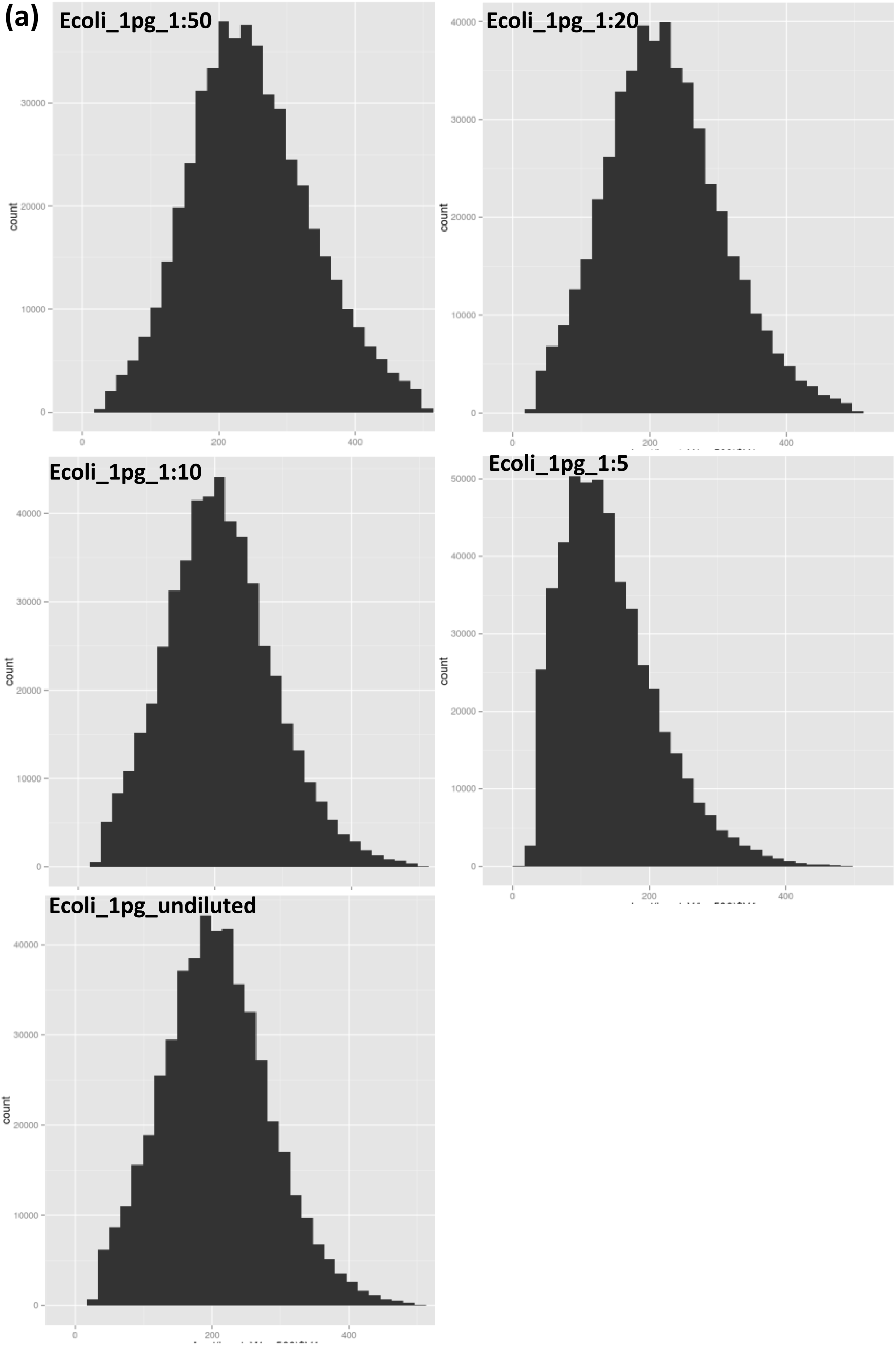
(a)
Ecoli_1pg_1:50
Ecoli_1pg_1:20
Ecoli_1pg_1:10
Ecoli_1pg_1:5
Ecoli_1pg_undiluted
count

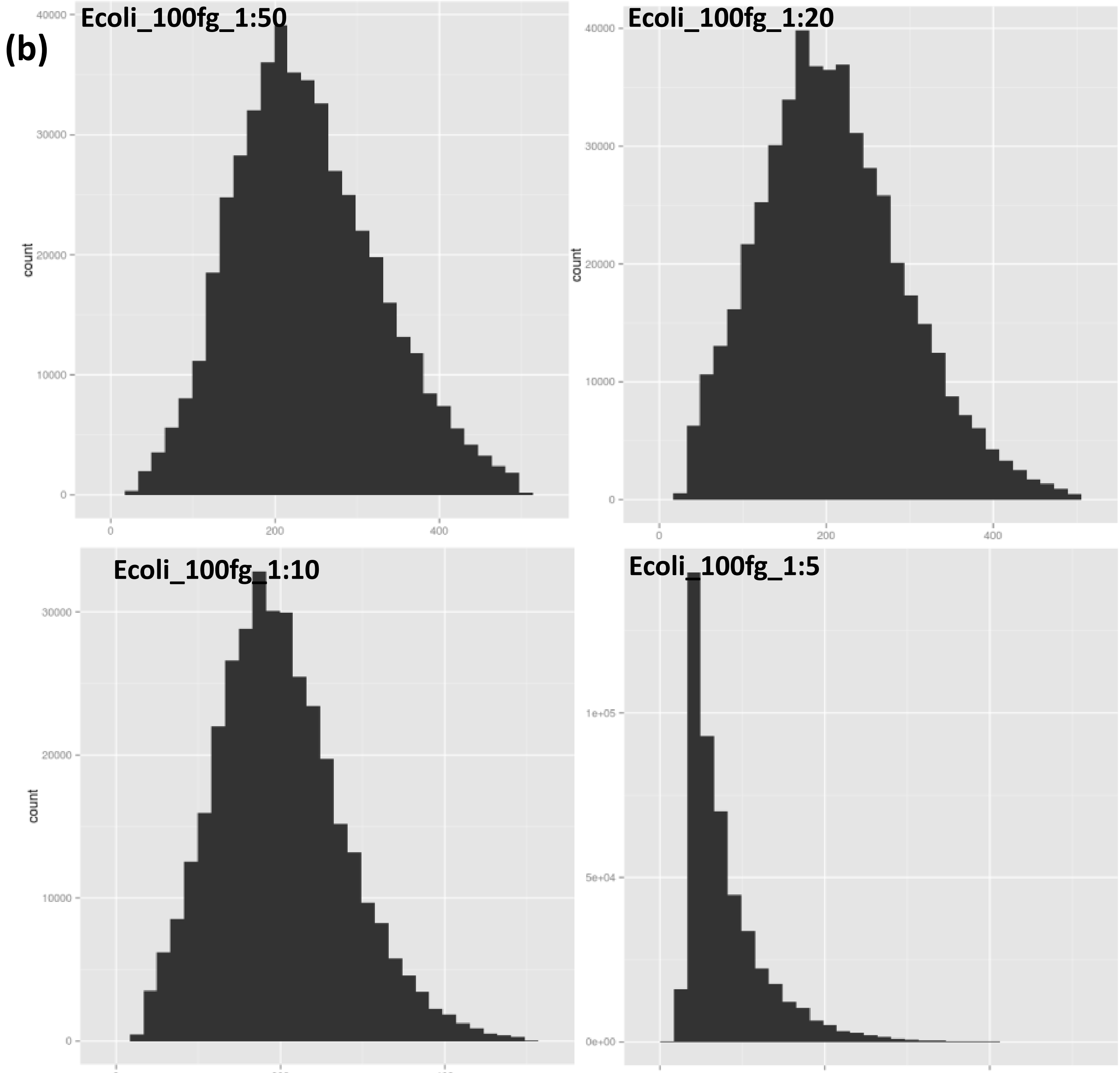

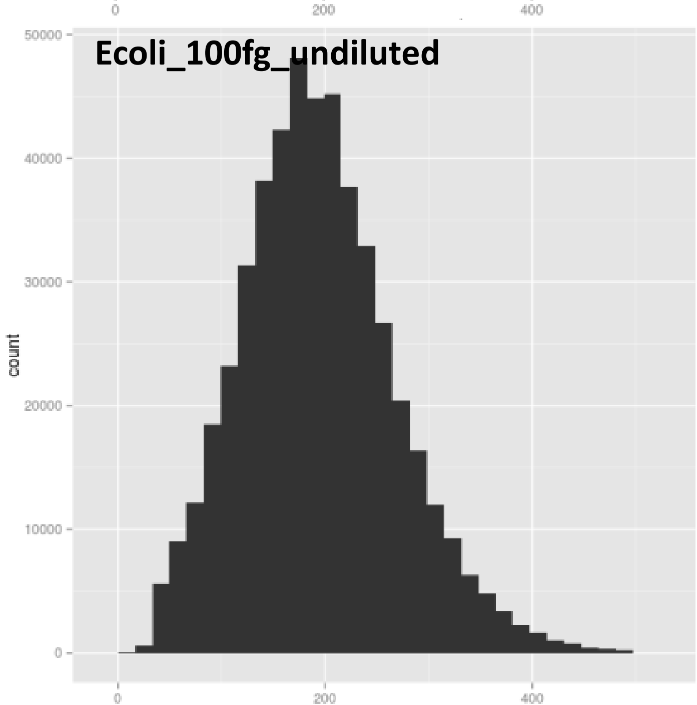

Figure S3 | Insert size distribution of *E. coli* low input libraries, using different ATM dilutions. Dilutions of the ATM (amplicon tagmentation mix) range from 1:50 to down to 1:5, and are compared to the undiluted ATM. (a) shows the insert size distribution for the low input DNA 1pg *E. coli* libraries and (b) for the 100fg *E.coli* libraries.

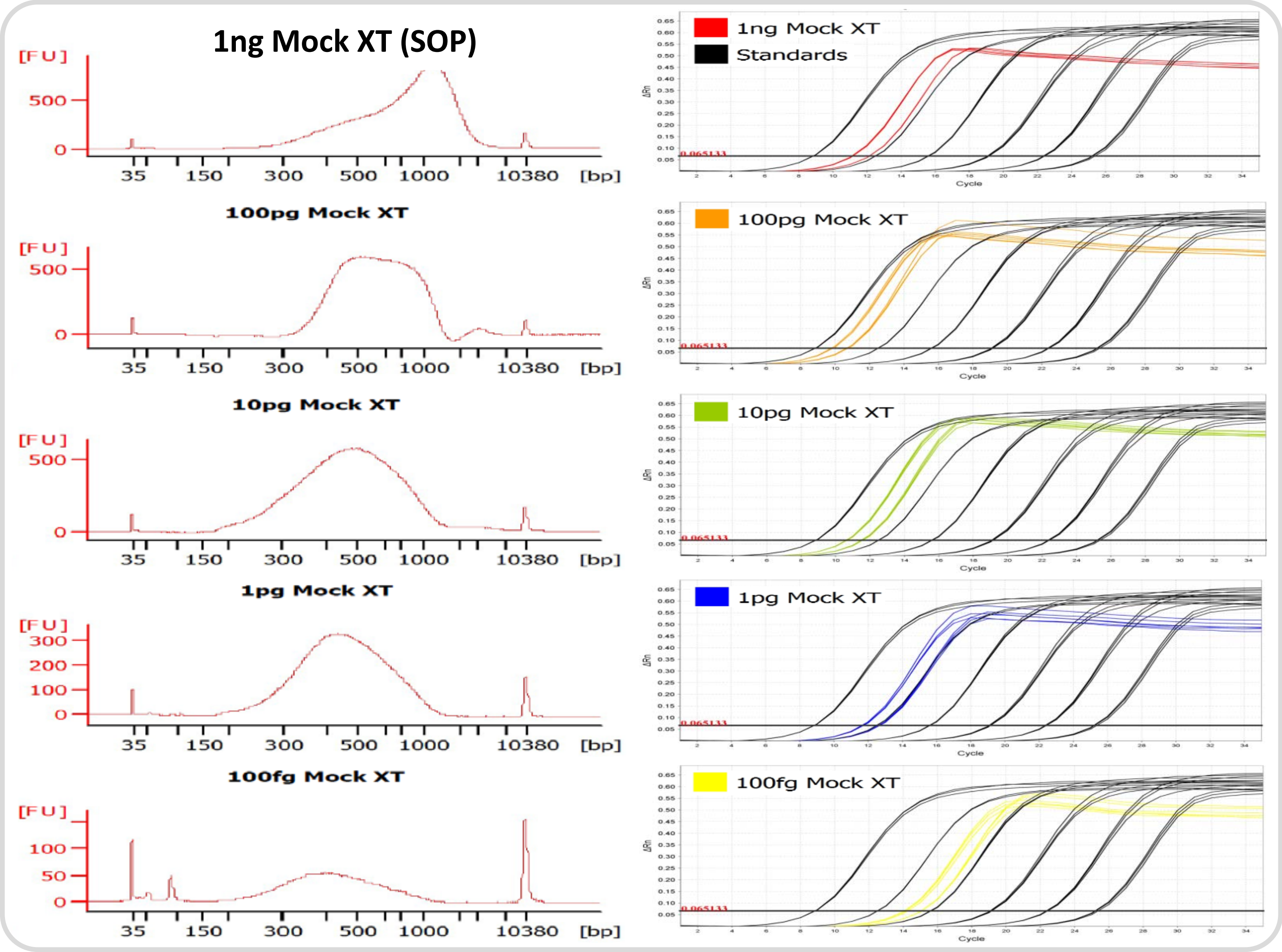

Figure S4 | Examples of Bioanalyzer and qPCR profiles for different low input DNA concentrations. The sequencing libraries were created from the microbial mock community samples.

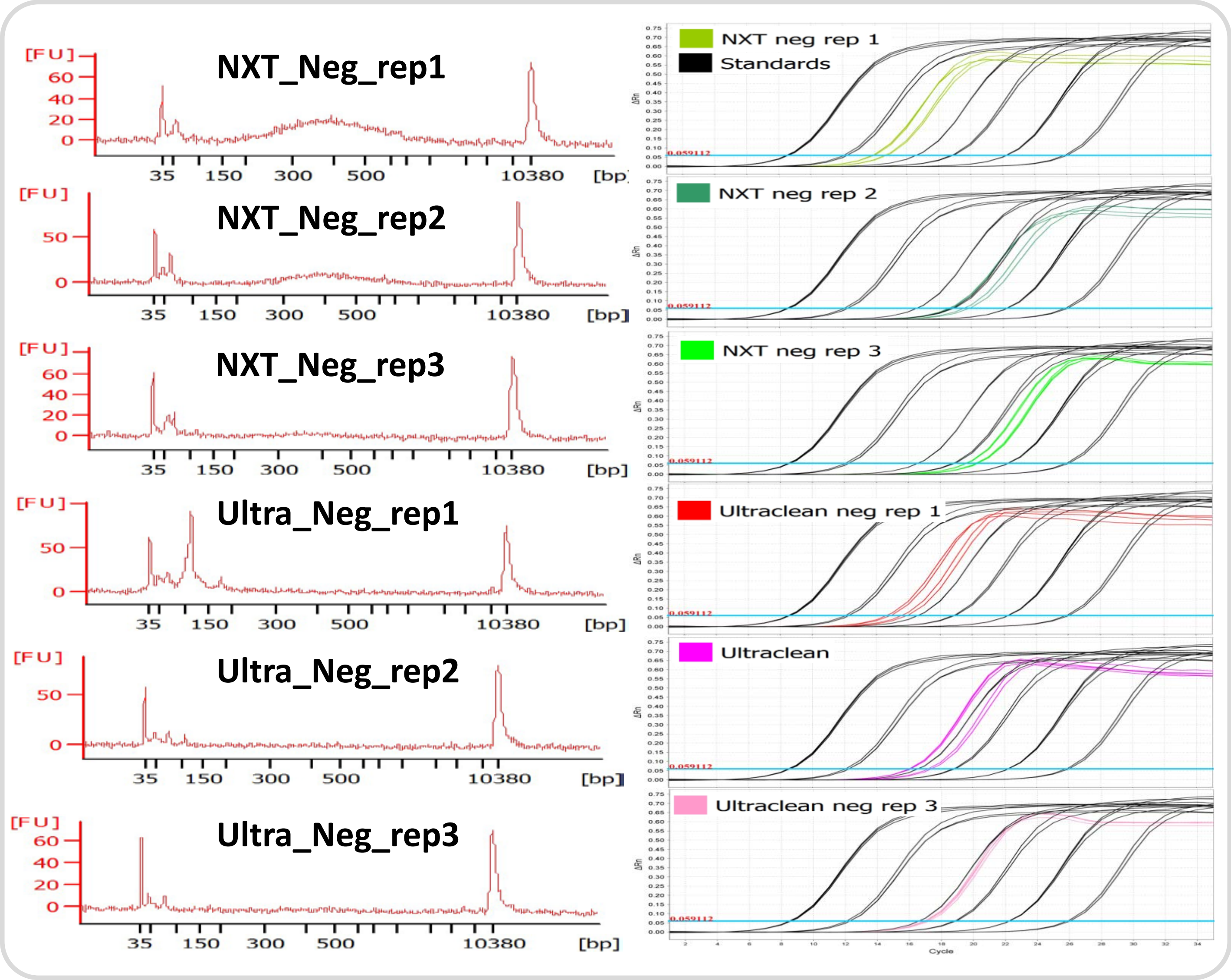

Figure S5 | Bioanalyzer and qPCR profiles of negative controls. Negative controls were detectable via the Bioanalyzer and/or qPCR assays and were therefore sequenced. Negative controls were carried out by substituting ddH_2O for input DNA for library construction (NEXT_Neg) and for DNA extraction followed by library construction (Ultra_Neg).

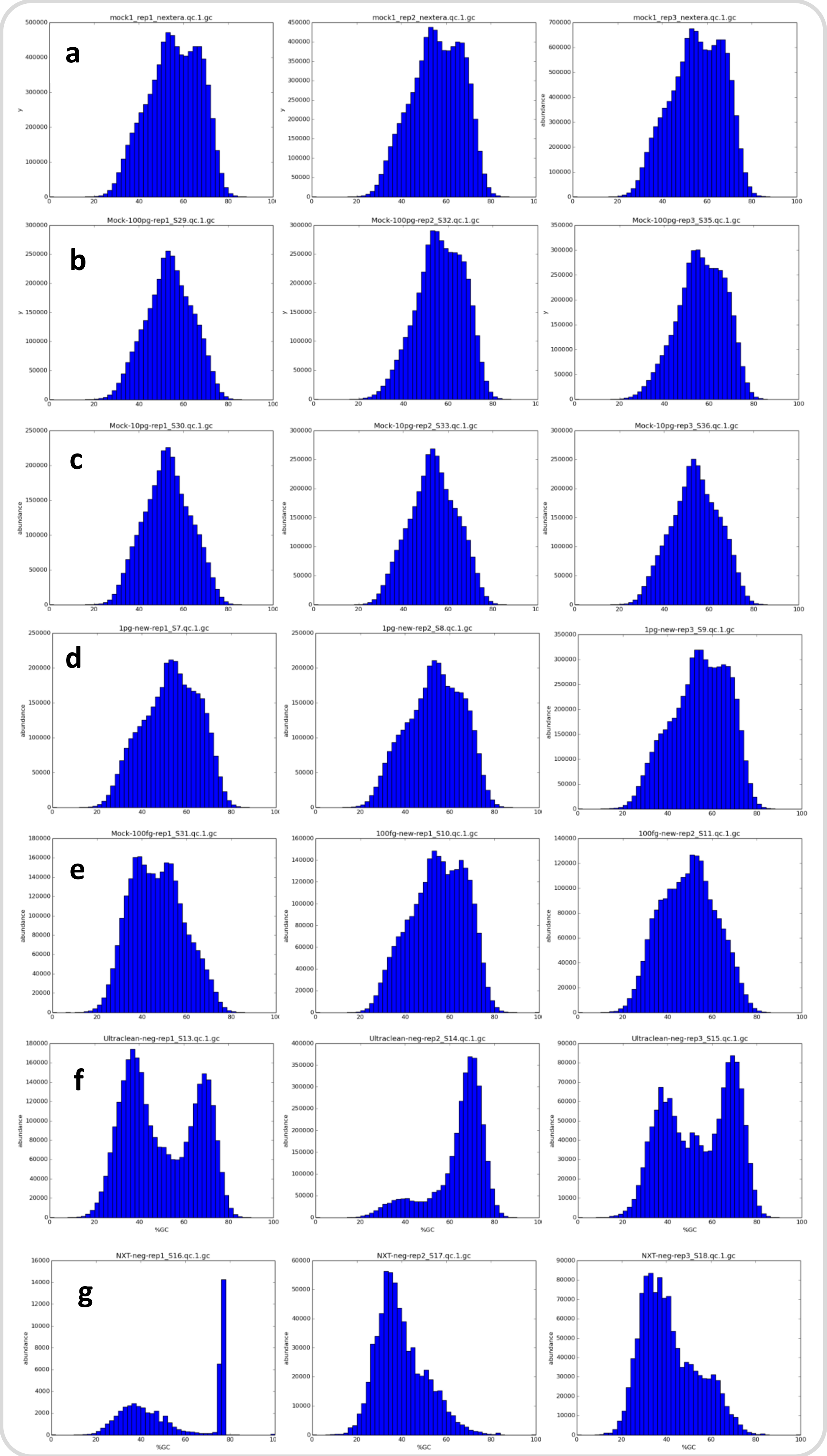

Figure S6 | Mock community read GC content. Shown are (a) 1ng SOP, and the low input libraries (b) 100pg, (c) 10pg, (d) 1pg, (e) 100fg, and the negative controls (f) Ultra-clean DNA extraction kit plus library preparation kit, (g) library preparation kit.

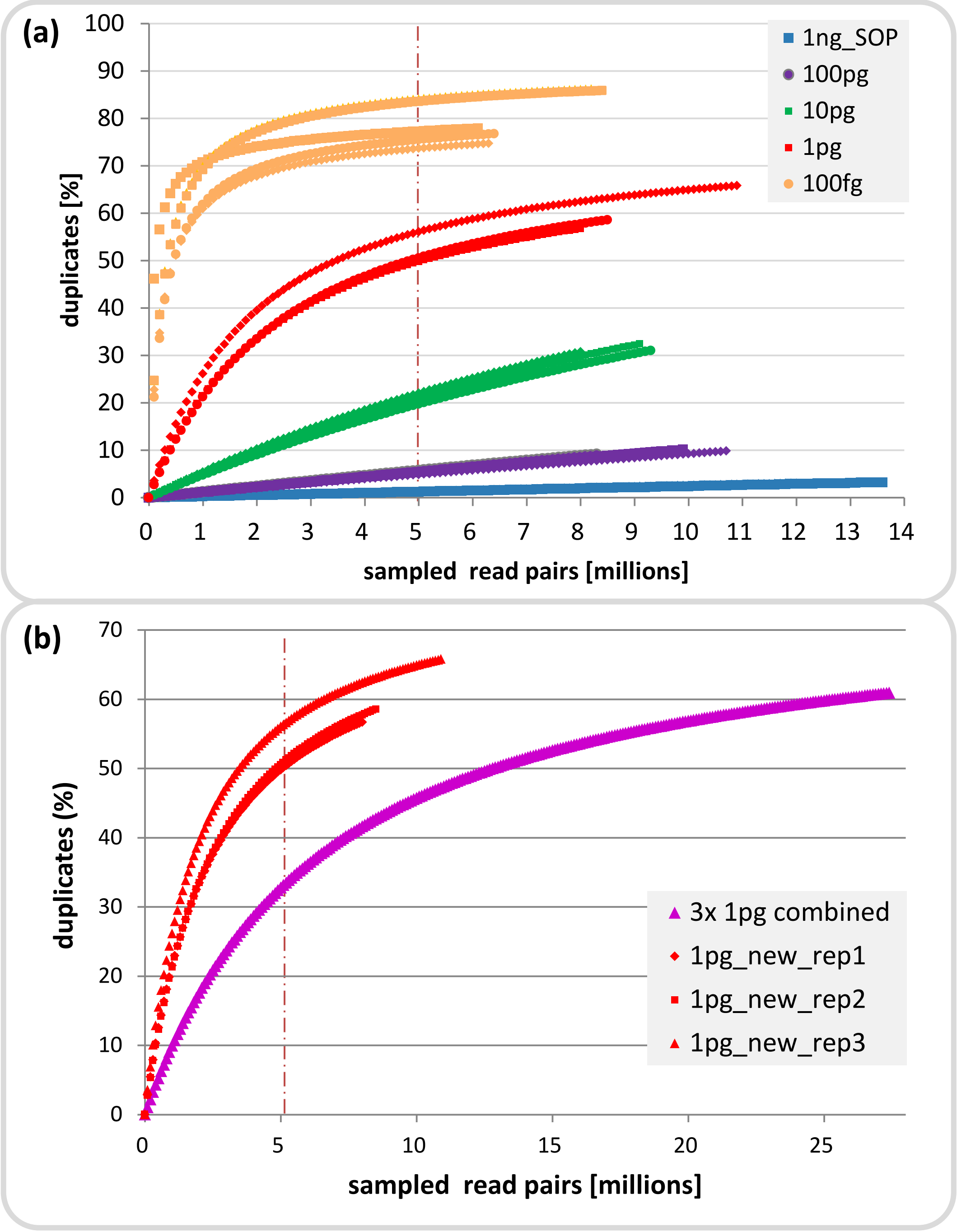

Figure S7 | PCR read duplicates. (a) Reads identified as duplicates in the mock community SOP and low input DNA libraries. (b) Observed decrease of read duplicates when combining three replicates of the 1pg libraries. The sequencing depth of 5 million read pairs, used to compare libraries, is accentuated by a dashed line

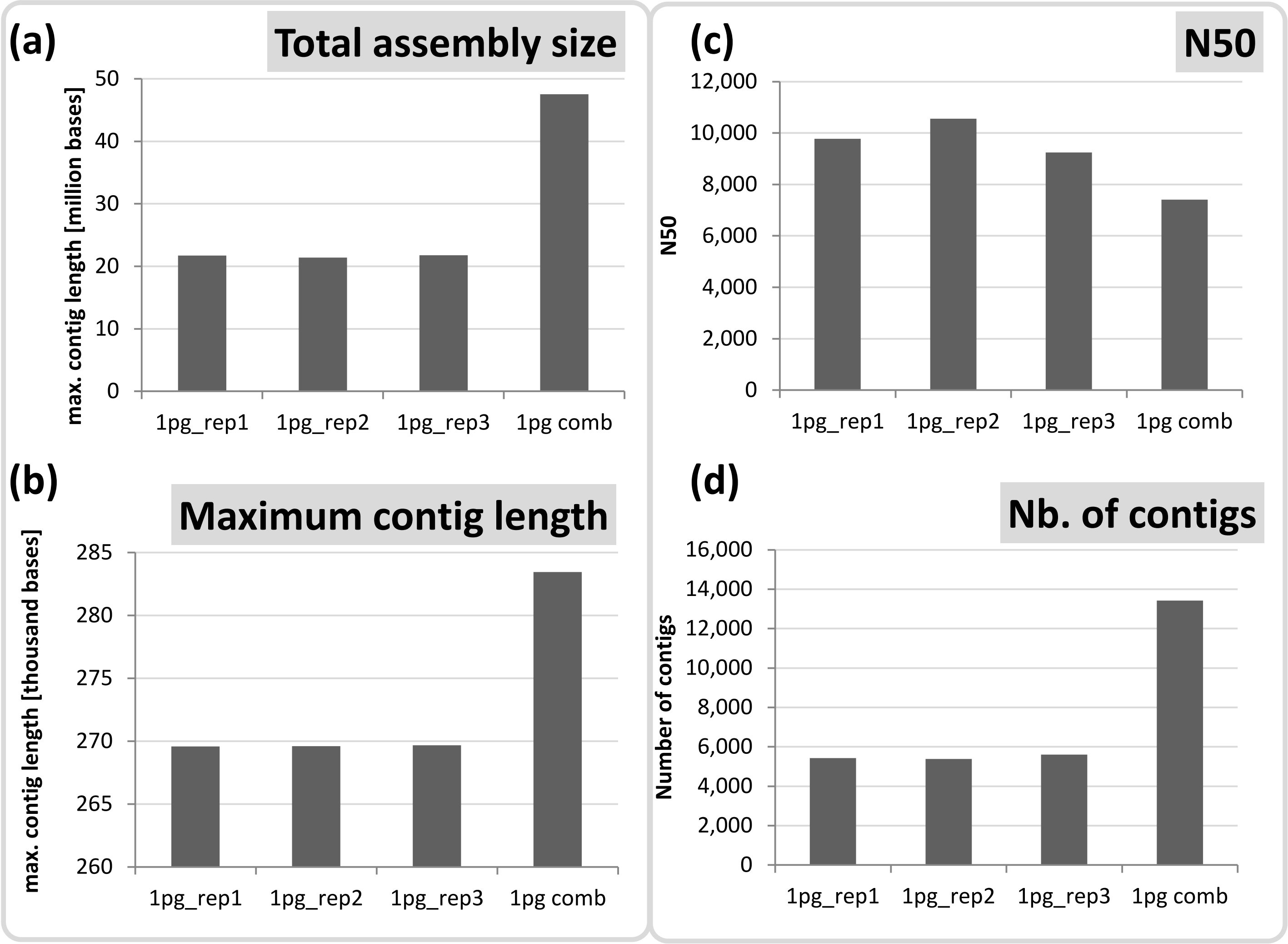

Figure S8 |Assembly of multiple replicates. Three replicates form the 1pg low input DNA libraries were assembled separate (rep 1,2,3) and combined (comb). **(a)** Total assembly size, **(b)** Maximum contig length, **(c)** N50 , and **(d)** number of contigs

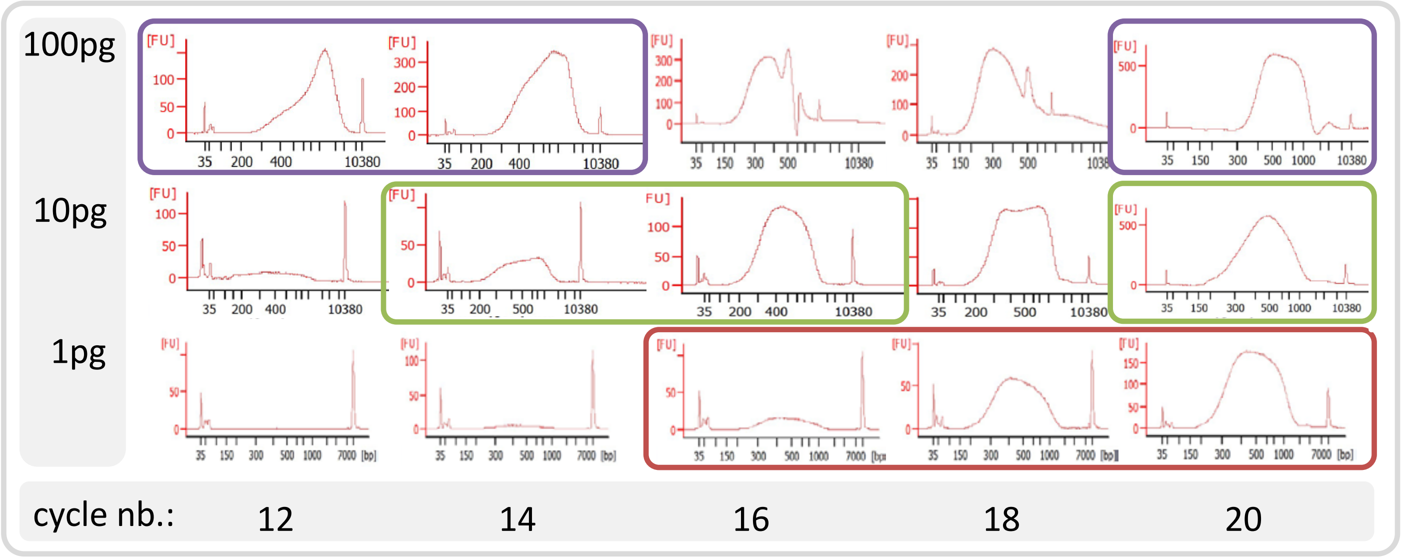

Figure S9 Bioanalyzer detectability thresholds as a function of limited cycle PCR. The range of tested limited PCR cycles starts at 12 cycles (as used in the SOP) and increases in two-step intervals to 20 cycles (used in our modified protocol). The Bioanalyzer plots are shown for each tested cycle number using 100pg, 10pg, and 1pg input DNA. The coloured boxes indicate the libraries above the Bioanalyzer detectability thresholds, which were selected for sequencing. ATM dilutions of 1:10 were used for all libraries.

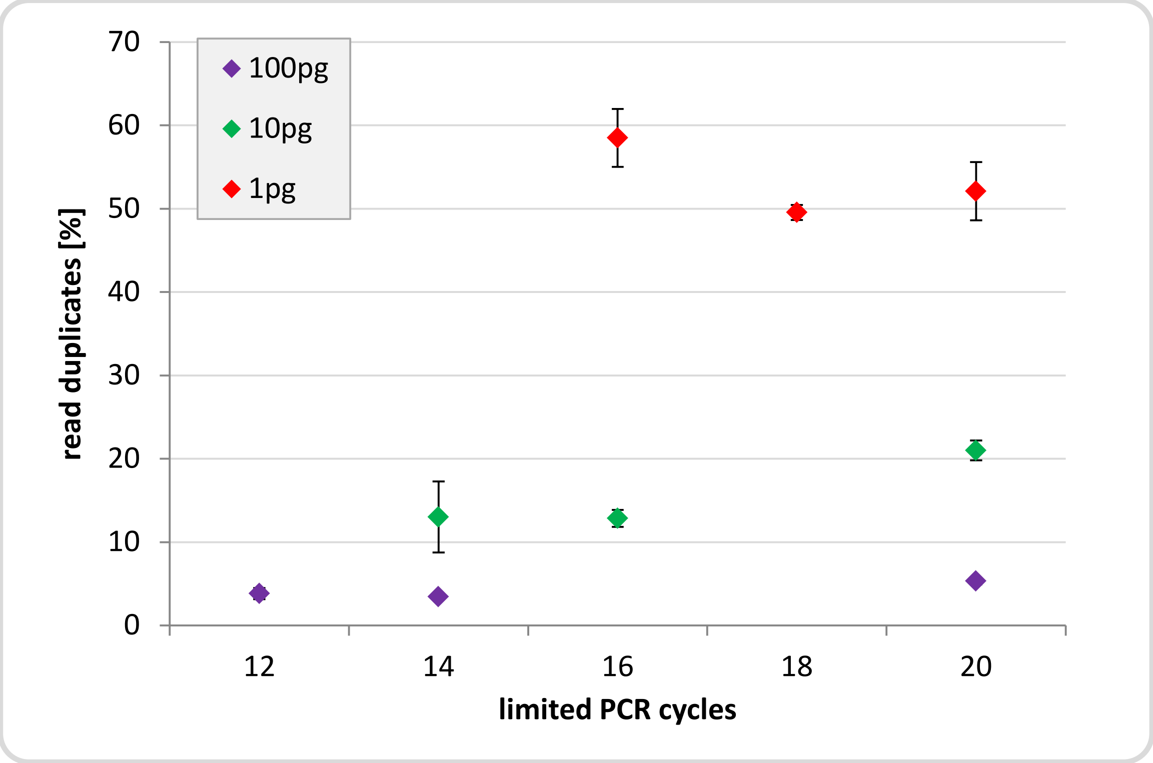

Figure S10 | Read duplicates and limited PCR cycles. The relation between the precent of read duplicates (y-axes) and the number PCR cycles (x-axes) reveals no decrease of read duplicates with lower cycle numbers.

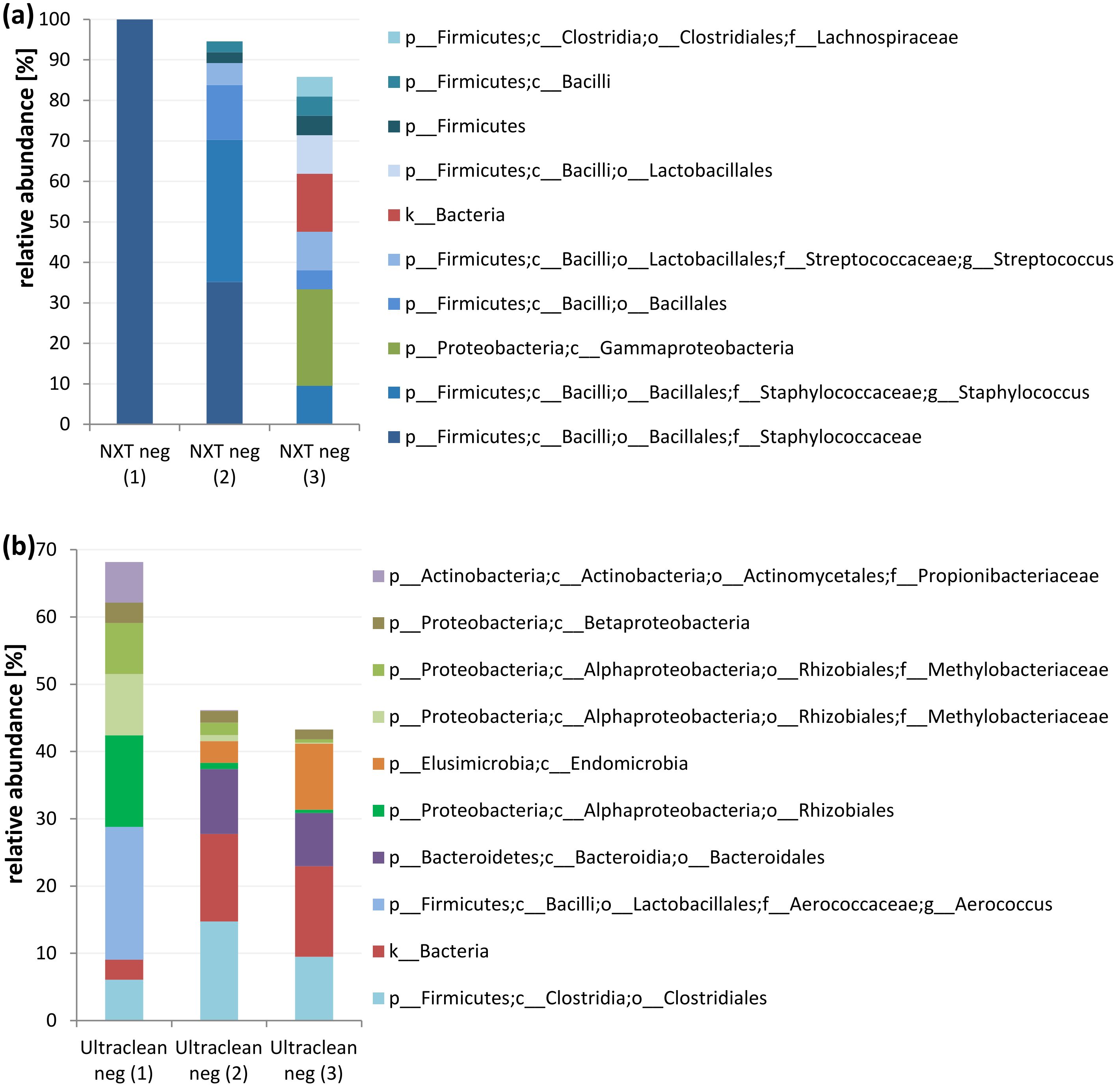

Figure S11 | Taxonomic profile of unassigned reads. Unmapped reads not mapping to our reference database which includes the mock community, human, phiX and *Methylobacterium aerolatum,* as shown in Figure 2, were profiled via short read aligner graftM utilizing the 16S rRNA package. **(a)** Taxonomic profile of unmapped Nextera XT negative controls (NXT neg) replicates, showing top ten hits, **(b)** taxonomic profile of top ten hits of DNA extraction plus Nextera XT negative controls (Ultraclean neg) replicates.) Please not that the color schemes are different between the graphs.

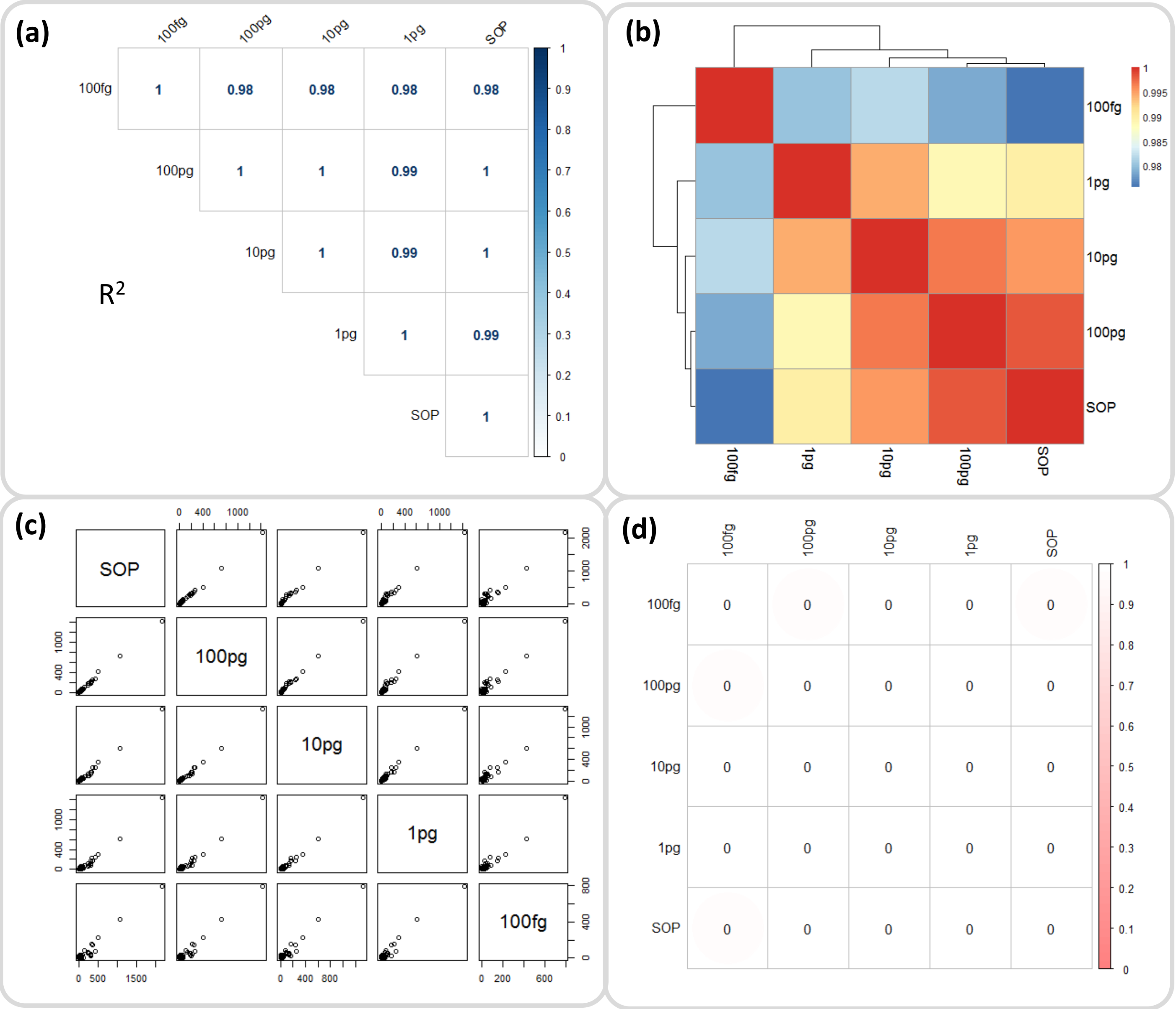

Figure S12 | Mock community 16S rRNA-based taxonomic profiles. Correlations are shown for the SOP and the low input DNA libraries. **(a)** Pearson correlation coefficient (R^2) , **(b)** correlation matrix, **(c)** correlation plot, and **(d)** significance test of the observed correlations. The applied cut-off was a maximum >10 reads per OTU.

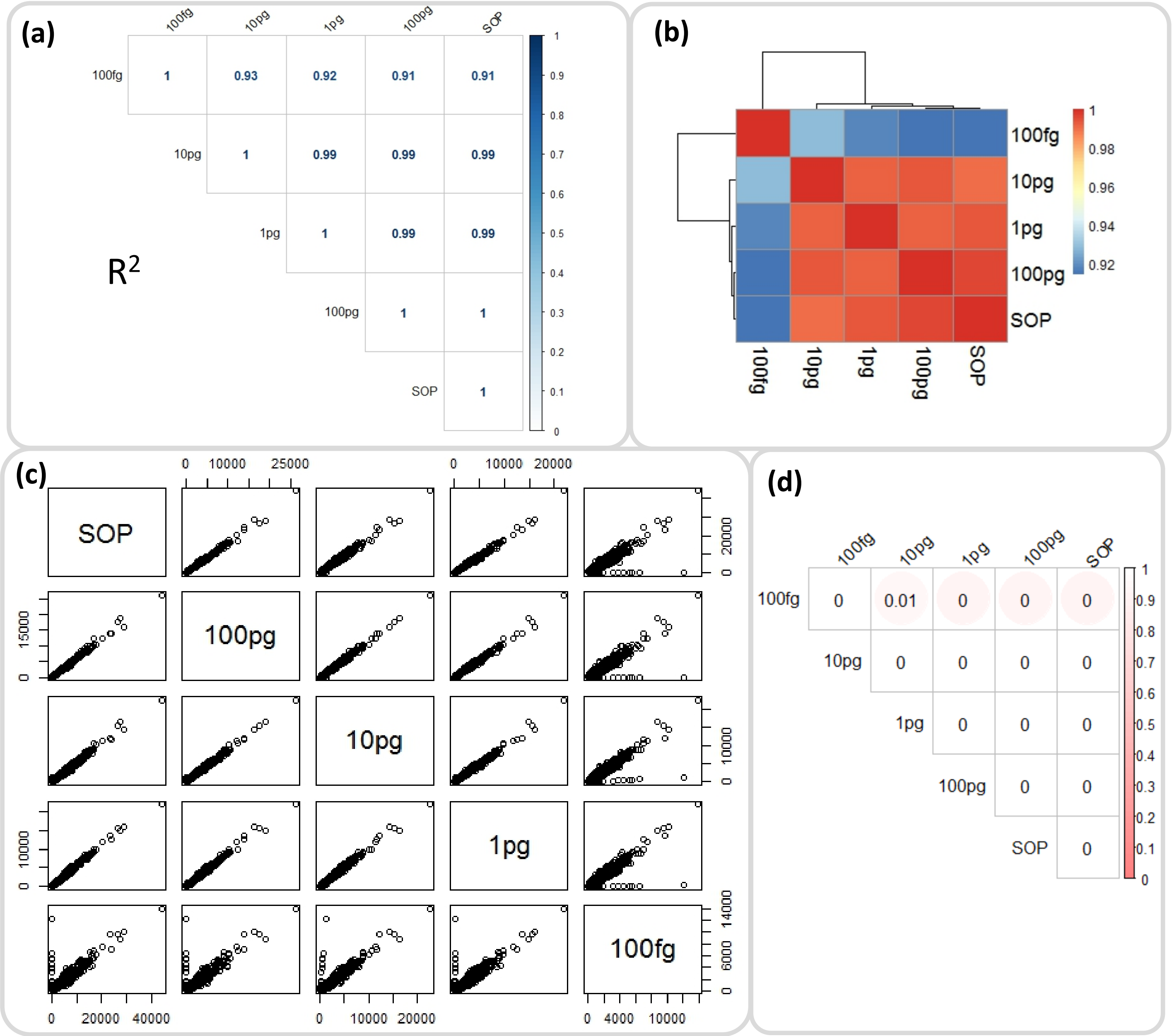

Figure S13 | Functional profile analysis. Correlations are shown for the SOP and the low input DNA libraries. **(a)** Pearson correlation coefficient (R^2), **(b)** correlation matrix, **(c)** correlation plot, and **(d)** significance test of the observed correlations. Reads were aligned with DIAMOND (BLAST X mode) against the KO (KEGG Orthology) database and a cut-off maximum >500 was applied to the resulting output table.

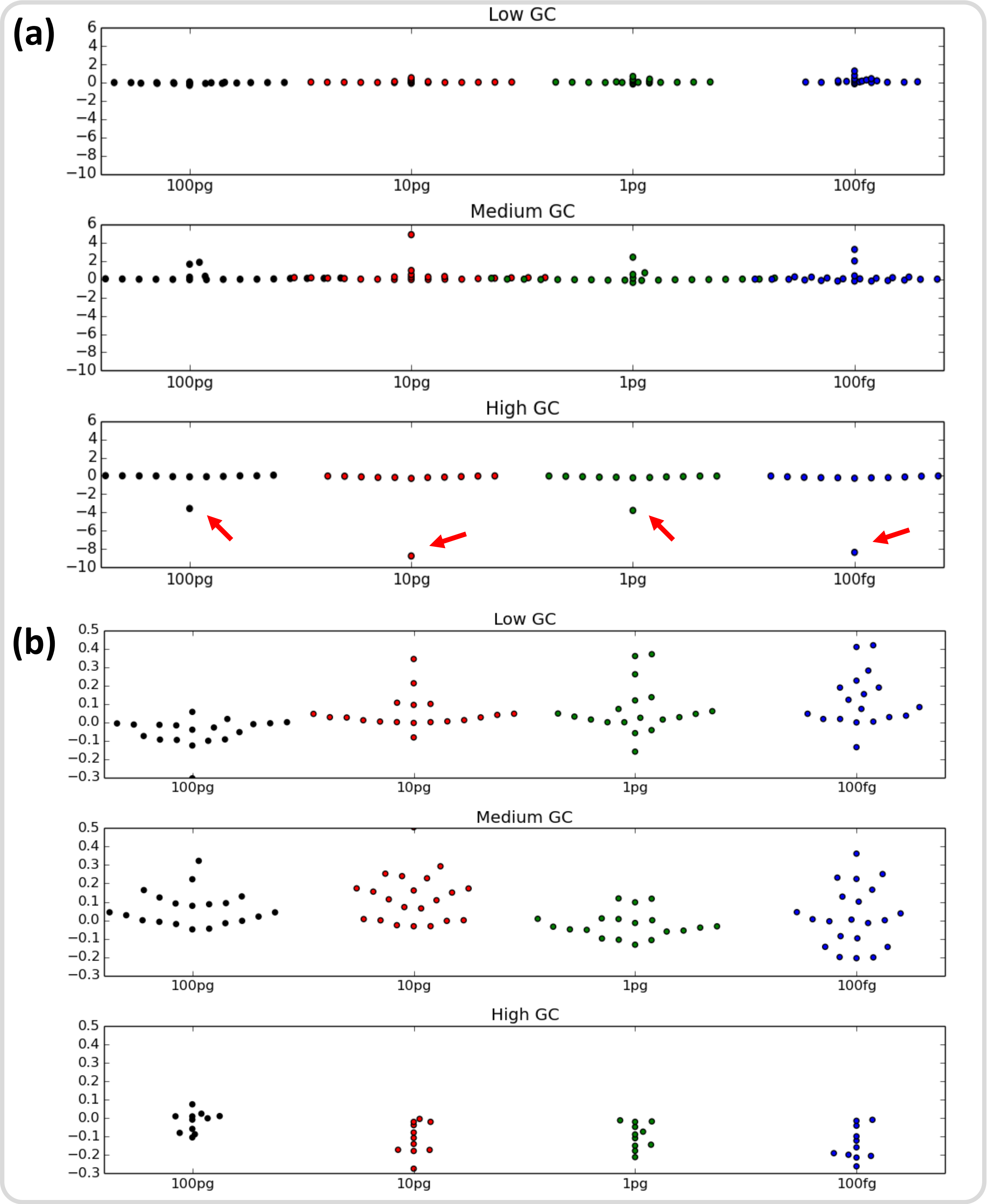

Figure S14 | Differences in relative abundance of the low DNA libraries to the 1ng SOP library, grouped by GC content. Each data point represents the average GC content of a mock community member, whereby the organisms are grouped in three categories: Low GC (<40% GC; top graph), medium GC (41-60% GC; middle graph), high GC (>61% GC, bottom graph). (a) All 54 mock community members were included. The strongest outlier was found in the High GC group (-4 to-8%; red arrows) and was identified as *Burkholderia sp*. (67% GC), the second most abundant member in our mock community. (b) Organisms producing outliers above +/- 1% were not included. The Y-axes shows the differences in relative abundance, based on read mapping, of the mock community members by comparing the low input libraries to the 1ng SOP.

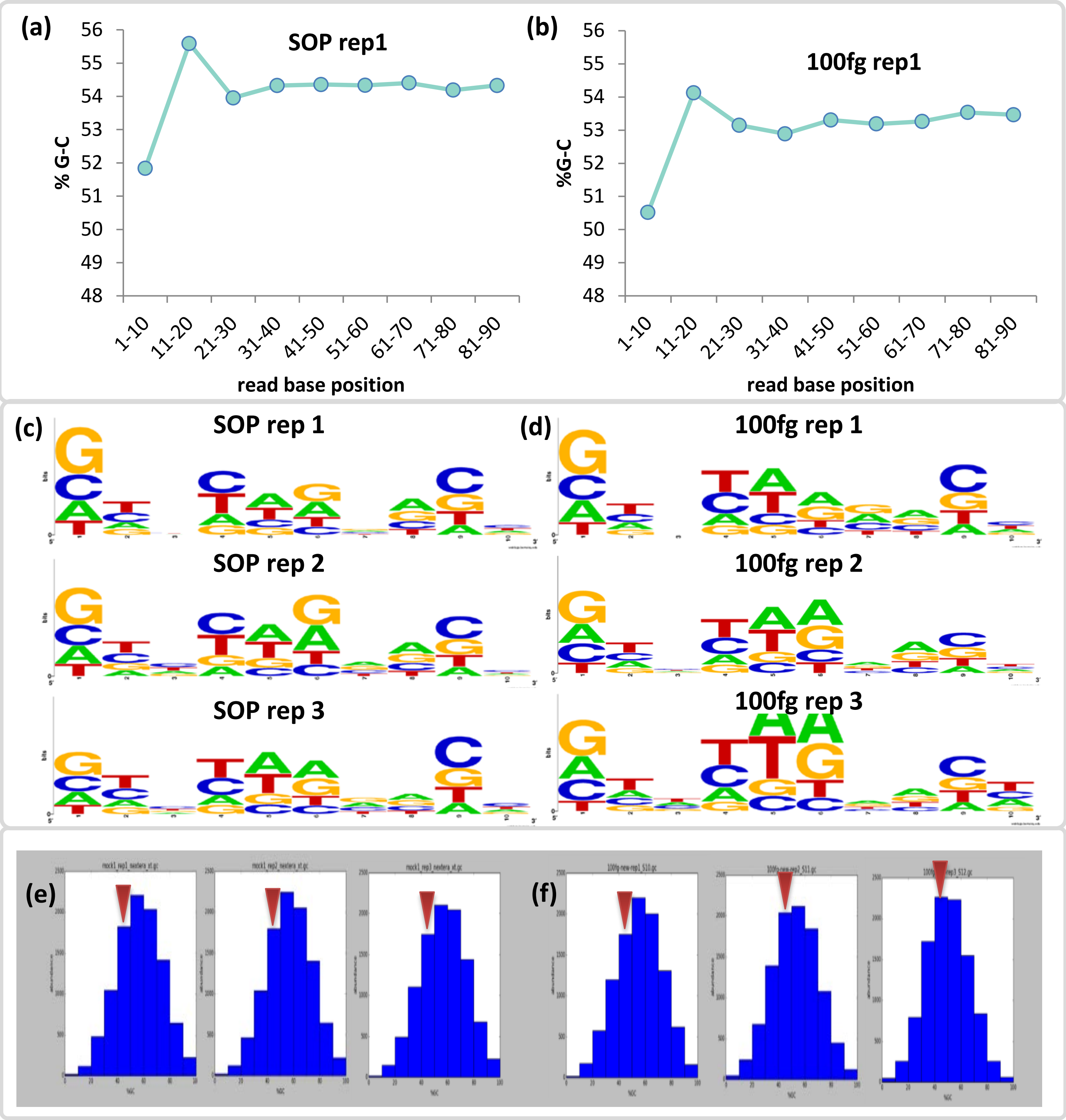

Figure S15 | Transposase insertion sites. Average GC content for reads of **(a)** a 1ng SOP and **(b)** a 100fg low input library. Reads were randomly subsampled to 10,000 and the average %GC was analyzed in 10 base segments from the start of the read to base position 90. Sequence logo analysis of **(c)** 1ng SOP and **(d)** 100fg low input libraries. The first 10 bases of a random subset of 1000 reads were analysed, showing a slight preference for insertion sites starting with Guanine (G). Each logo consists of stacks of symbols, one stack for each position in the sequence. The overall height of the stack indicates the sequence conservation at that position, while the height of symbols within the stack indicates the relative frequency of each amino or nucleic acid at that position. The even heights of the symbols indicate that the distribution is near random on most position. However the minute differences can be used to extract the consensus target site for the SOP libraries which is GTNYARRACN, and for the 100fg libraries which is GTNTAARACN showing a slightly higher AT frequency. The GC content of transposase insertion sites of **(e)** 1ng SOP and **(f)** 100fg low input libraries. The first 10 bases of a random subset of 1000 reads were analyzed for library replicates. Note the GC category 40-60 %GC (red triangle) is more pronounced in some of the 100fg input libraries.

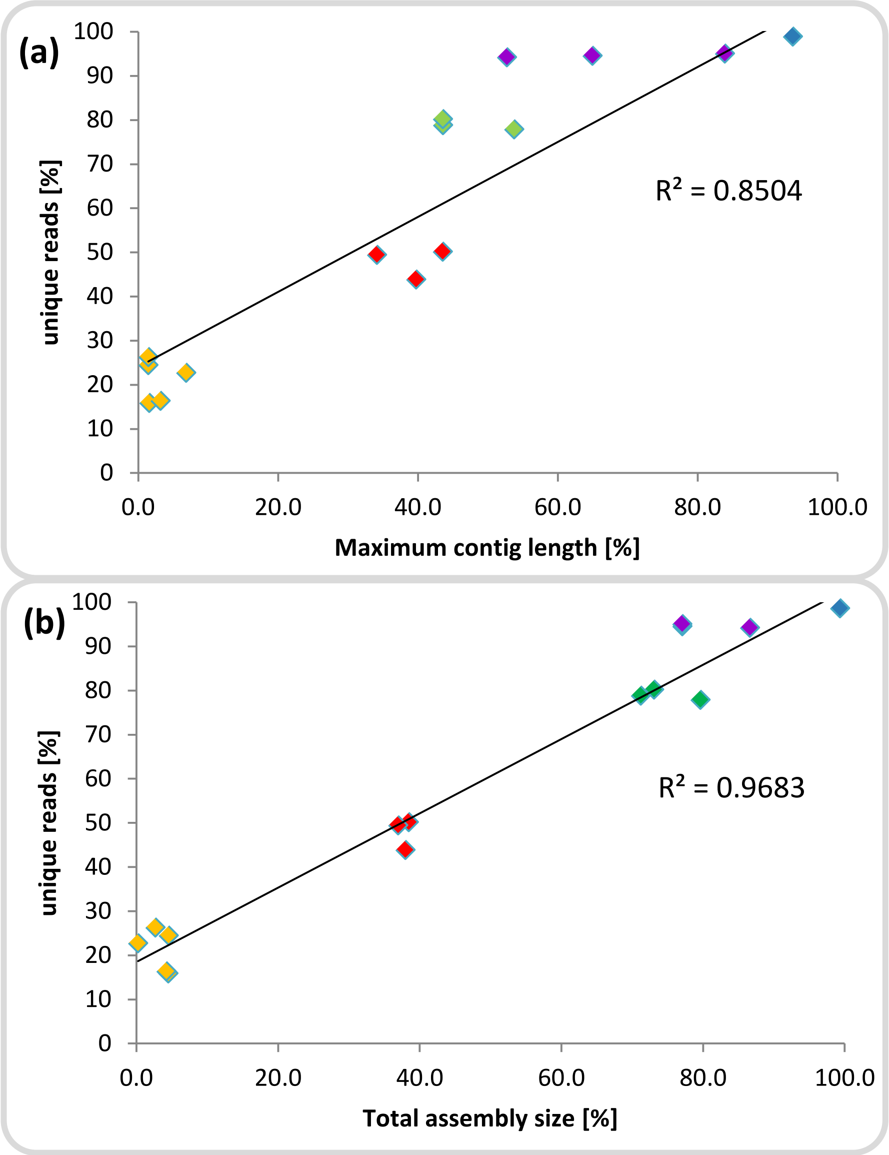

Figure S16 | Correlations between the percentage of unique reads and assembly statistics. **(a)** Maximum contig size, **(b)** total assembly size. Read files were subsample to 5 million reads pairs prior assembly and only contigs ≥ 1kb were included in the analysis. The Pearson correlation coefficient and a linear trend line is given for each correlation.

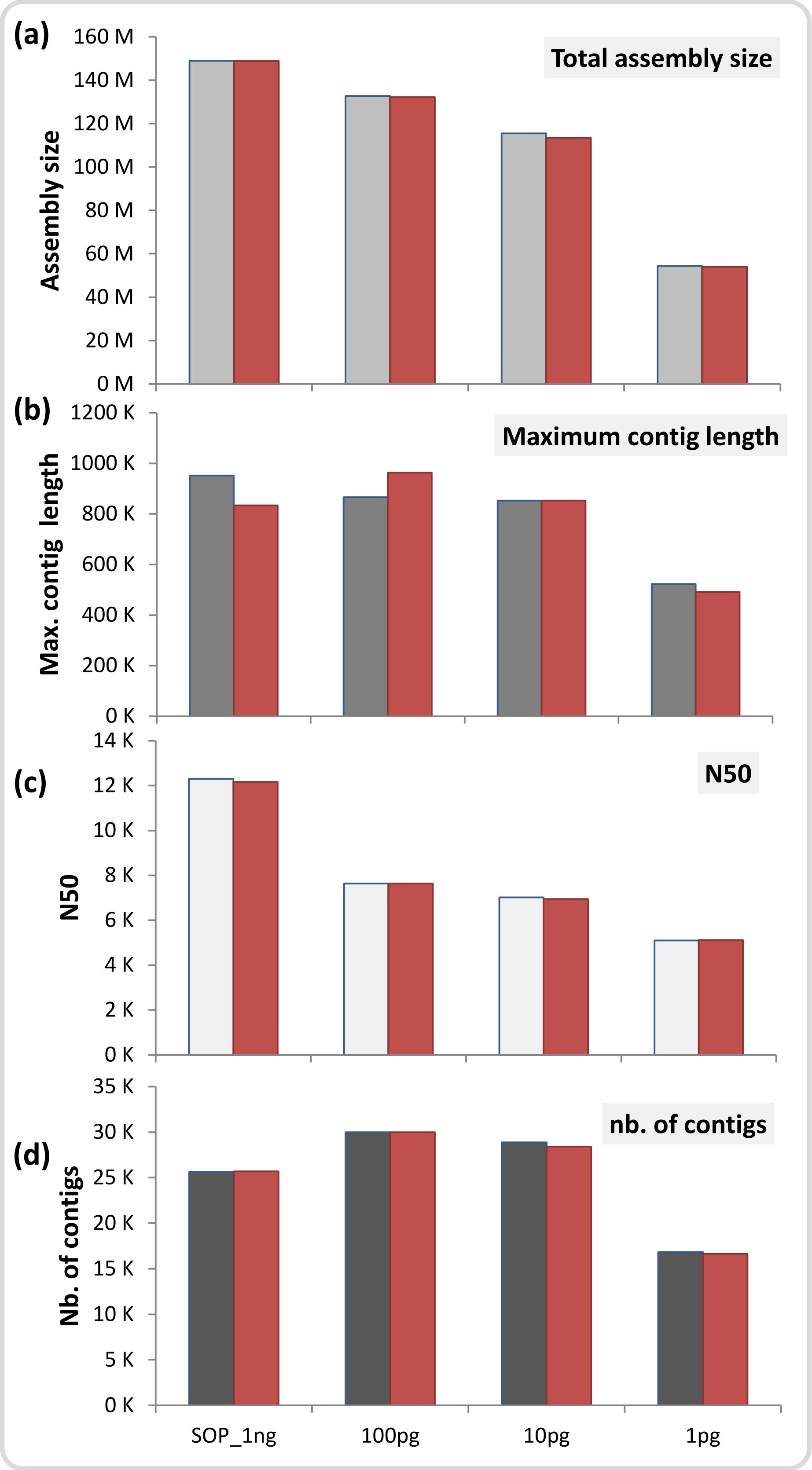

Figure S17 | Mock community assembly statistics utilizing 50 million reads. **(a)** Maximum contig size, **(b)** total assembly size, **(c)** number of contigs, and **(d)** N50 of the SOP and low input mock community libraries. The assembly statistics is shown for all reads (gray shaded bars) and without read duplicates (red bars). Read files were subsample to 50 million reads. Only contigs ≥ 1kb were included in the analysis.

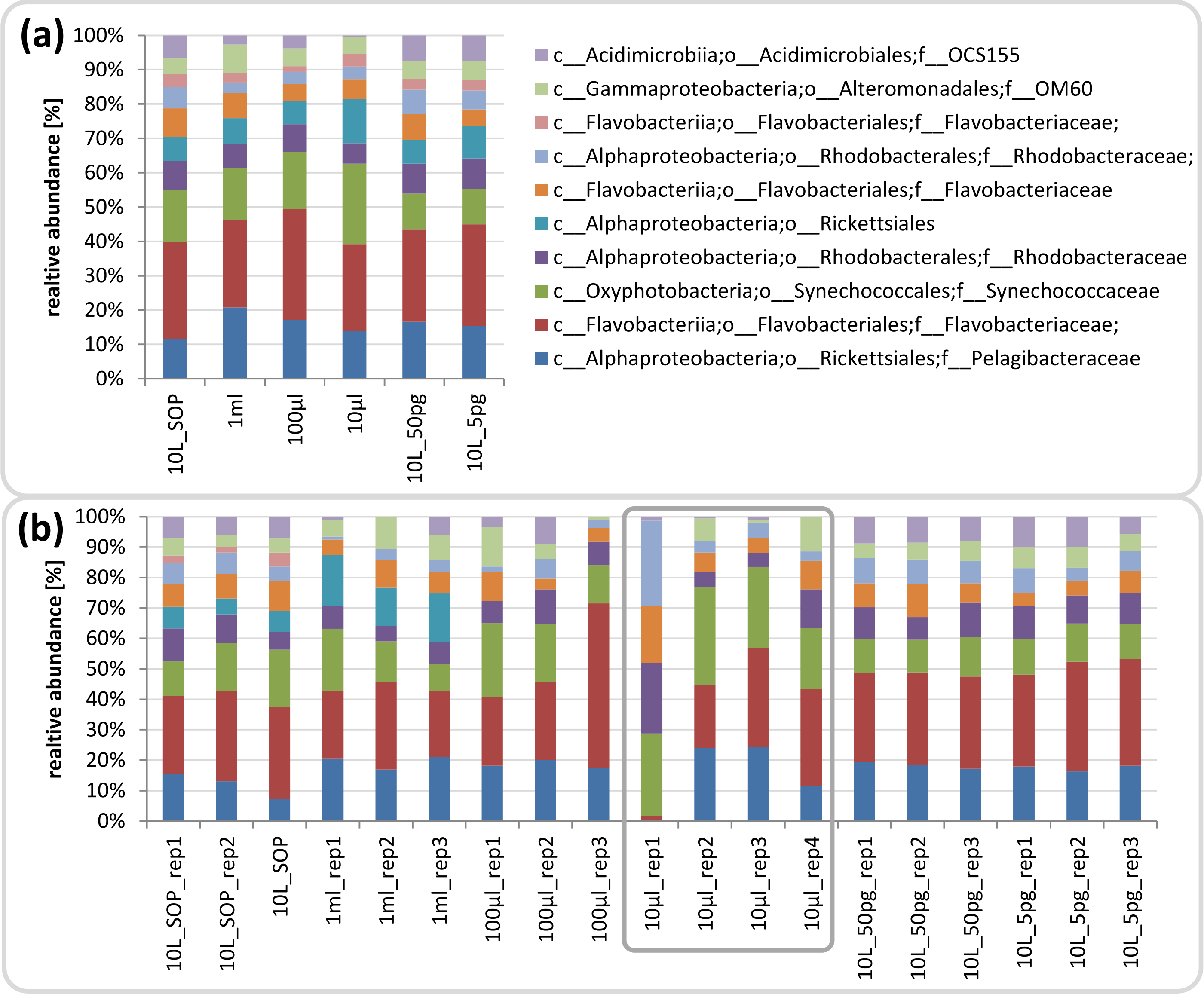

Figure S18 | Relative abundance profiles of marine microbial samples. Bacterial OTUs were assigned based on 16S rRNA gene sequence detection of shotgun sequencing reads (graftM; see Methods). The relative abundance is shown comparing the 10 most abundant OTUs. Plastid sequences belonging to microbial eukaryotes and the contaminant *Methylobacterium* were not included in the analysis. (a) Average number of reads per OTU for each sample type, (b) number of reads per OTU for each replicate. Note the increased variation among replicates of the 10µl sample (dark gray box).

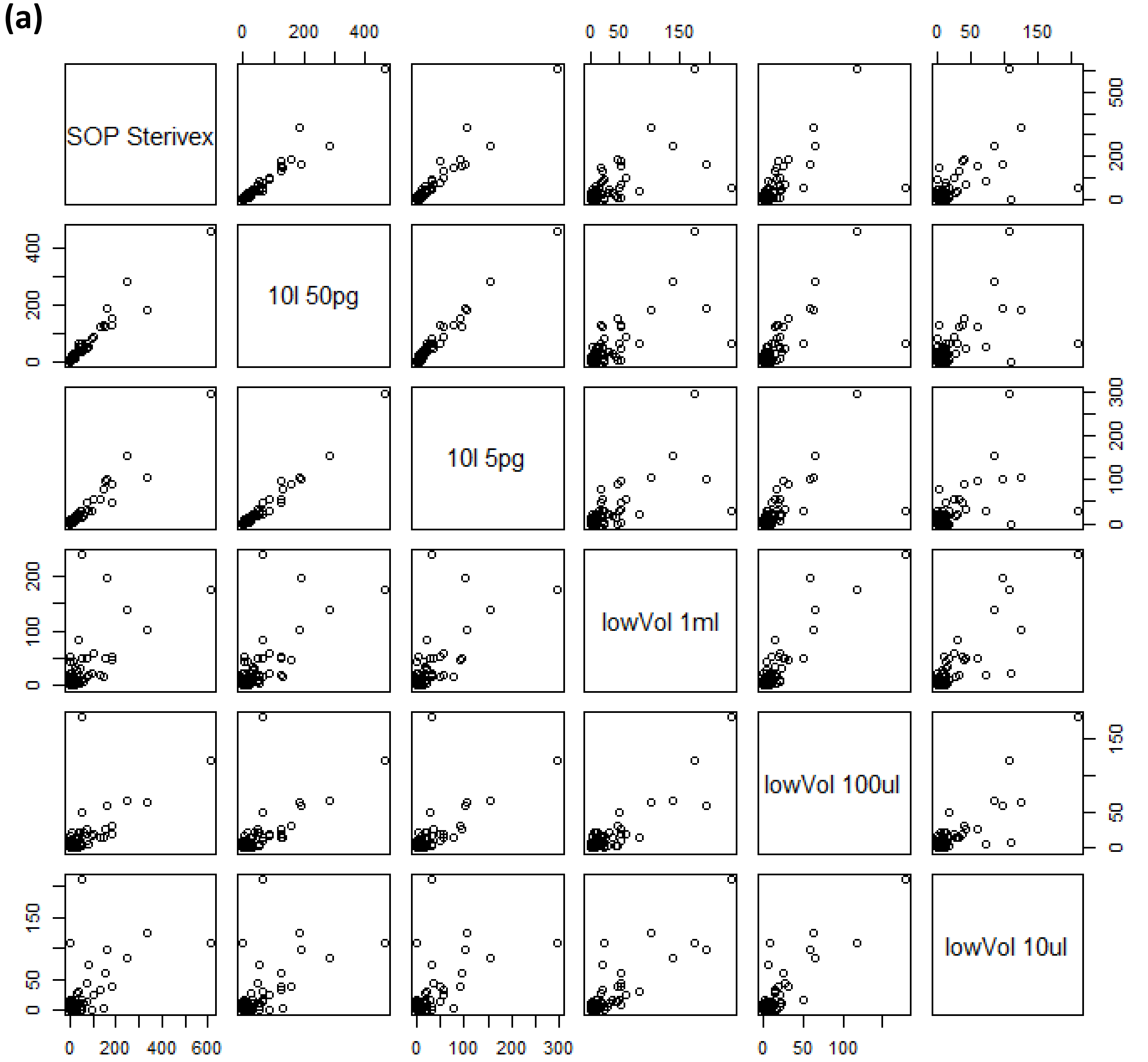

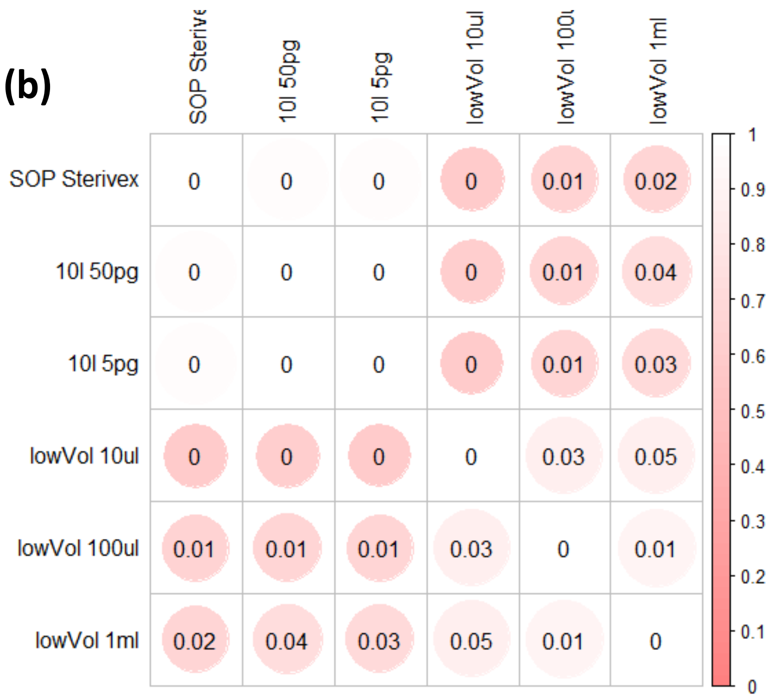

Figure S19 | 16S rRNA gene based profile analyses of marine samples. (a) Correlation plots, (b) significance tests for the marine SOP, the low volume filter dilution, and the low input DNA libraries.

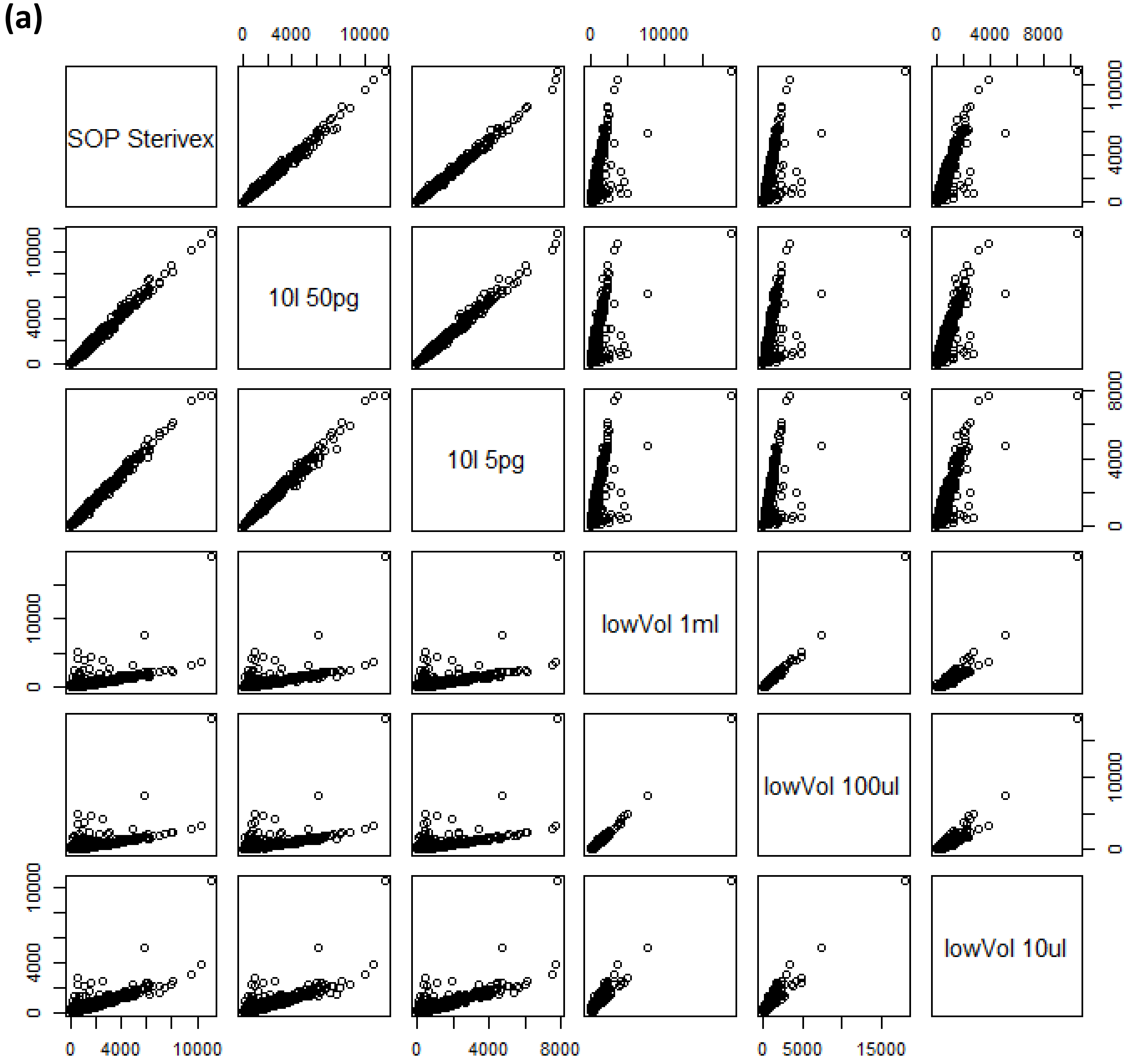

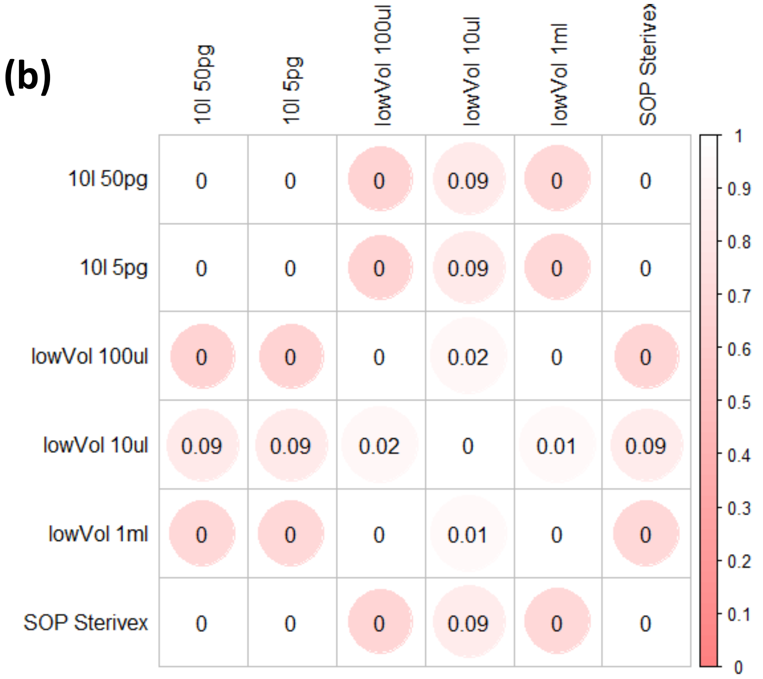

Figure S20 | KO-based functional profile analyses of marine samples. (a) Correlation plots, (b) significance tests for the marine SOP, the low volume filter dilution, and the low input DNA libraries. Per definition, a p-value <0.05 indicates that the correlation is significant.

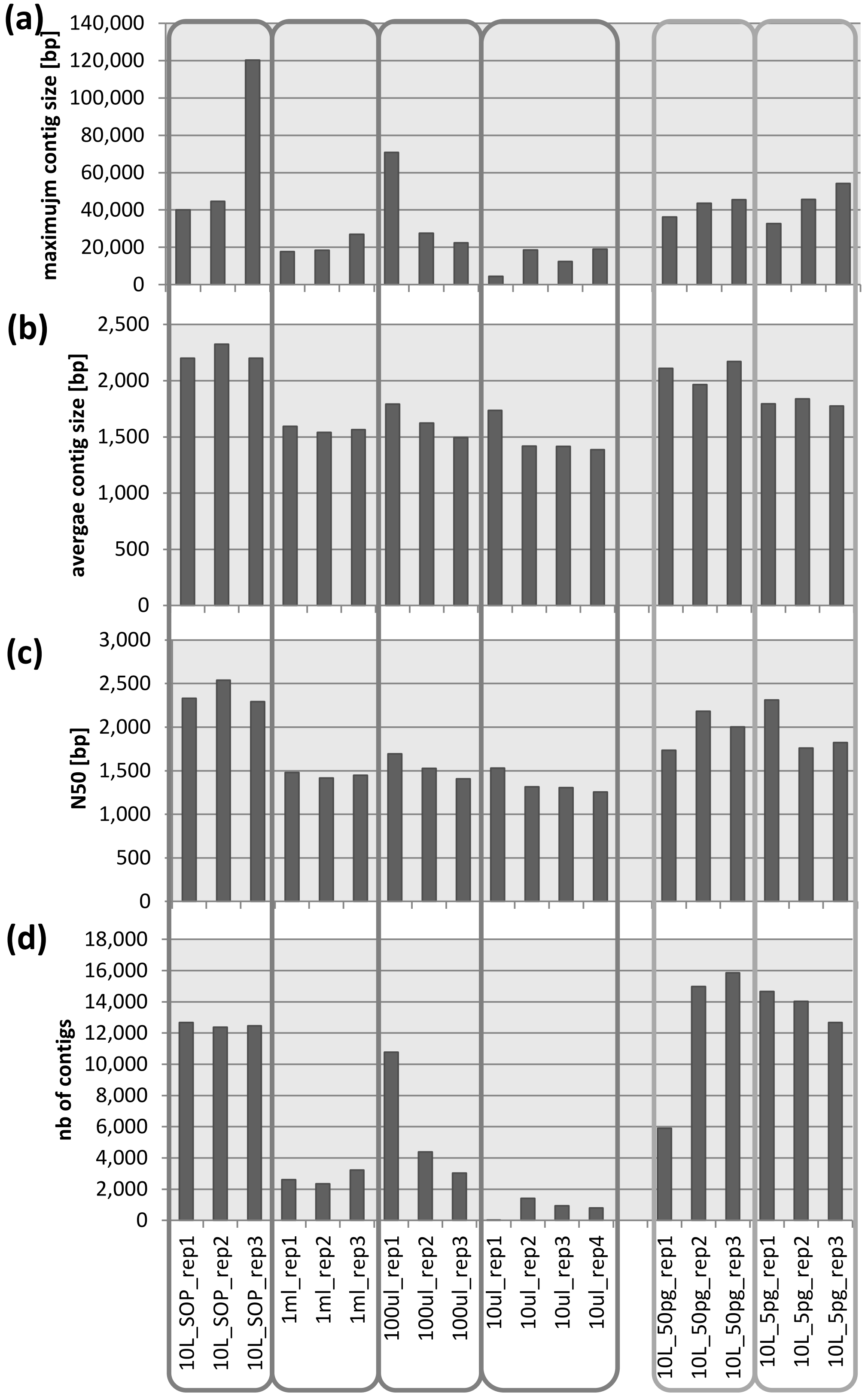

Figure S21 | Marine surface sample assembly statistics. (a) Maximum contig size, **(b)** average contig size, **(c)** N50 and **(d)** N number of contigs of the SOP and low input mock community libraries. Read files were subsample to 5 million read pairs.. Only contigs ≥ 1kb were included in the analysis.
